# Growth Stages and Inter-Species Gut Microbiota Composition and Function in Captive Red Deer (*Cervus elaphus alxaicus*) and Blue Sheep (*Pseudois nayaur*)

**DOI:** 10.3390/ani13040553

**Published:** 2023-02-04

**Authors:** Yao Zhao, Jia Sun, Mengqi Ding, Romaan Hayat Khattak, Liwei Teng, Zhensheng Liu

**Affiliations:** 1 College of Wildlife and Protected Areas, Northeast Forestry University, Harbin 150040, China; 2 Liaoning Wildlife Protection and Epidemic Disease Monitoring Center, Dalian 116013, China; 3 State Key Laboratory of Urban Water Resource and Environment, School of Environment, Harbin Institute of Technology, Harbin 150090, China; 4 Institute of Zoology, Guangdong Academy of Sciences, Guangzhou 510260, China; 5 Key Laboratory of Conservation Biology, National Forestry and Grassland Administration, Harbin 150090, China

**Keywords:** gut microbes, captivity, 16S rRNA gene sequencing, red deer, blue sheep, growth stage

## Abstract

**Simple Summary:**

Gut microbiota play a significant role in the diet digestion and health of the host. Many zoos provide research sites for biological scientists to reintroduce animals, especially those facing extinction. Therefore, to prevent the extinction of wild animals, captive wild animals have become one of the ways to protect threatened taxa. Monitoring the structure and function of the gut microbiota of captive animals can help elucidate whether animals have adapted to their artificial environment, which is one of the key issues in wildlife conservation. This study investigated the gut microbiota of blue sheep and red deer at different growth stages and inter-species. The result shows gut microbiota of blue sheep and red deer will change to some extent in different growth stages, but the dominant flora remains stable. In blue sheep and red deer, the gut microbiota differ significantly between species. In addition, several potentially pathogenic microbial communities were identified based on the current findings. The study highlights key gut microbiota across species and ages.

**Abstract:**

Blue sheep and red deer, second-class key protected animals in China, are sympatric species with a high degree of overlap of food resources in the Helan Mountains, China. Previous studies with blue sheep and red deer in nature have shown that their physiology is closely related to their gut microbiota. However, growth stages and changes occurring in these species in captivity are still unknown. Thus, *16S rRNA* gene sequencing was used to explore diversity, composition and function of the gut microbiota in these two animal species. The diversity and structure of the gut microbiota in captive blue sheep and red deer changed at different growth stages, but the dominant microbiota phyla in the gut microbiota remained stable, which was composed of the phyla Firmicutes, Bacteroidetes and Verrucomicrobia. Moreover, gut microbiota diversity in juvenile blue sheep and red deer was low, with the potential for further colonization. Functional predictions showed differences such as red deer transcription being enriched in adults, and blue sheep adults having a higher cell wall/membrane/envelope biogenesis than juveniles. Microbial changes between blue sheep and red deer at different growth stages and between species mainly depend on the abundance of the microbiota, rather than the increase and absence of the bacterial taxa.

## 1. Introduction

In animal hosts, the functions of the gut microbiota often influence nutrient metabolism, intestinal health, immunity and several other aspects, especially in captive animals, for which the ability to adapt is crucial [1,2,3,4,5,6,7,8]. The gut of animals becomes colonized by microbes after parturition, and gut microbiota play an important role in maintaining host homeostasis [9]. It is known that composition and diversity of the gut microbiota fluctuate dynamically throughout growth stages [10]. Some studies have proved that gut microbiota change continuously with growth and development [11,12,13], and gut microbiota become more similar with age [14]; among them, it was noted that the gut microbiota in feces of children was significantly lower than that of adults [15]. Due to different diets and physiological levels at different ages [16], changes in intestinal microbiota with different growth stages can also reflect the health level of animals. Furthermore, considerable differences in the composition of gut microbial communities have been identified among different animal species, and diversity in gut bacteria plays an important role in the growth and health of animals [17].

Blue sheep (*Pseudois nayaur*), belonging to the family Bovidae, and red deer (*Cervus elaphus alxaicus*), belonging to the family Cervidae [18], are widely distributed species of ungulates commonly found in the Helan Mountains, China. Both species are considered endangered ruminants whose populations have been observing a sharp decline due to global climate change and intensive land use. Thus, these animals are listed as national key second-class protected animals in China, being categorized as least-concern species by the International Union for Conservation of Nature (IUCN) [19,20]. In nature, blue sheep and red deer are sympatric species with a relatively high niche overlap of food resource acquisition [21,22]. Based on the physiological characteristics of ruminants and the morphological characteristics of plants consumed by these animals, blue sheep and red deer are intermediates between concentrate selectors/browsers and roughage eaters/grazers, thus being classified as mixed feeders [23,24,25,26]. Moreover, due to differences in the digestive tract of these two species, different plant species or plant parts within a certain species can be consumed and coexist harmoniously [27].

In China, research on the gut microbiota of blue sheep and red deer at different growth stages in captivity is scarce. With the rapid development of society, a growing number of animals has been facing extinction threat; thus, captivity has been increasingly used to ensure protection and reintroduction, especially for endangered animals [28,29,30]. Ungulates are mostly reared in semi-enclosed outdoor facilities, which limits their exposure to microbes. However, captive breeding ensures that animals are provided with good living conditions for very stable growth and development. Nevertheless, captive animals might still be affected by diseases [29,31] and they may face more pressure due to limited space [32]. Monitoring the structure and function of the gut microbiota of captive animals can help elucidate whether animals have adapted to their artificial environment, which is one of the key issues in wildlife conservation [33]. Therefore, improving the environmental adaptability of wild animals in captivity ensures better survival and reproduction.

Thus, this present study aimed to explore both the intra- and inter-species diversity of the gut microbiota in captive blue sheep and red deer at different growth stages. In addition, intra- and inter-species differences in the composition of the gut microbiota and biomarkers of growth stages were explored. Furthermore, core bacteria were determined for each developmental stage and host, and the main functions of the identified core bacteria were analyzed. Collectively, the findings help to elucidate complex interactions between the host and the corresponding gut microbiota, thus providing clues to understand the ecology of the microbiota of captive blue sheep and red deer.

## 2. Materials and Methods

### 2.1. Study Design and Sample Collection

Fresh fecal samples were collected from animals in Zhongshan Park, Yinchuan, China, in August 2020. Zhongshan Park was established in 1993, aiming to ensure wildlife rescue and conservation for translocated populations. Blue sheep (n = 16, among which were 10 adults and 6 juveniles) and red deer (n = 16, among which were 10 adults and 6 juveniles). Adult animals were rescued from the Helan Mountains, China, and were not able to return to the wild after rehabilitation. Most juvenile animals were reared in captivity (zoos), and their progenitors were disabled animals that were rescued from the wild who otherwise would not survive (Table S1). The animals were reared in a semi-closed environment within an area of approximately 75 m^2^ and at temperatures ranging from 15–31 °C during summer (Video S1). Blue sheep and red deer at different growth stages were kept in similar semi-closed cages with 20–25 individuals per cage (Figure S1). All animals included in the present study were healthy and did not receive antibiotics. Finally, animals were fed twice daily with fresh corn, fresh alfalfa and a proportion of fermented silage, and had access to fresh water ad libitum. The collected fecal samples were stored at −80 °C.

### 2.2. DNA Extraction

Total genomic DNA was extracted from fecal samples using Qubit 3.0 DNA kit (Life Technologies Corporation, Gaithersburg, MD, USA) with CTAB/SDS method [34]. Library concentration and purity were determined using Qubit 3.0 fluorometer (Invitrogen, Carlsbad, CA, USA). The hypervariable V3-V4 region of the bacterial 16S rRNA gene was amplified using universal primers 338F (5’-ACTCCTACGGGGAGGAGCA-3’) and 806R (5’-GGACTACHVGGGTWTCTAAT-3’). PCR amplifications were conducted using Hieff^®^ Robust PCR Master Mix (Easen Biotechnology, SHH, CHN), and the PCR reaction system (30 μL of final volume) was composed of 15 μL of PCR Master Mix, 1 μL of primer F, 1 μL of index-PCR primer R, 0.2 μL of PCR product (30 ng) and 12.8 μL of H_2_O. PCR amplification conditions were as follows: 94 °C for 3 min; denaturation at 94 °C for 30 s, followed by annealing at 55 °C for 20 s and extension at 72 °C for 30 s for 20 cycles and a final extension at 72 °C for 5 min.

### 2.3. 16S rRNA Gene Sequencing

Purified PCR products were sent to Shanghai Sangon Biotech Co., Ltd. (Shanghai, China) for sequencing using an Illumina MiSeq platform. Paired-end 2 × 250 bp reads were generated, and N-base sequences were removed. Data files were converted into raw sequences (sequenced Reads) by base calling analysis, and results were reported as FASTQ files. Cutadapt [35] (http://cutadapt.readthedocs.io/en/stable/, accessed on 22 May 2021) was used to remove 3’-end sequencing primer adapter of Read1. PEAR [36] was used to merge paired reads into a single sequence according to the overlap between paired-end reads. PRINSEQ [37] was used to excise bases whose quality value was below 20 at the tail of the reads, with a 10-bp window.

### 2.4. Statistical Analysis

Sequencing results were clustered using Usearch [38,39] (http://www.drive5.com/usearch/, accessed on 22 May 2021). Obtained sequences with 97% similarity were divided into OTUs. Subsequently, 16S rRNA reads were classified against the SILVA [40] RNA database (http://www.arb-silva.de, accessed on 22 May 2021) using RDP classifier [41]. Annotation for each representative sequence was conducted with mothur algorithm in R software (version 3.6.0).

Alpha- and beta-diversity indices were calculated for all samples using QIIME2 version 1.7 [42] (https://view.qiime2.org/, accessed on 24 May 2021). Alpha-diversity metrics, including Chao1 and ACE indices, were calculated using Student’s *t*-test and Kruskal–Wallis test. Wilcox test was used to test differences in abundance levels between two sample groups. Non-metric multidimensional scaling (NMDS) was used to analyze differences in species diversity between samples. Anosim was used to analyze differences in microbial community structure among sample groups. ANOVA was used to analyze the effects of different growth stages and species on blue sheep and red deer. Linear discriminant analysis (LDA) Effect Size (LEfSe) [43] was used to determine significance among differences in the abundance of different microbial species, considering LDA score as 2. NMDS, rarefaction curve and rank abundance analyses were performed using the “ggplot2” package in R software (Lucent Technologies Co., Ltd., NJ, USA) to assess the degree of variability in the gut microbiota and sample size.

### 2.5. Gut Microbial Community Composition and Co-Occurrence Network Construction

Following the method proposed by Stegen et al., βNTI/RCbray values were used to quantify the variation in phylogenetic composition between samples [44]. For different growth stages, each sample was clustered 999 times by probability. βNTI values greater than +2 indicated that the observed difference in community composition was the result of deterministic selection, also called heterogeneous selection [45]; values between −2 and +2 indicated values referring to a random population set, also called dispersal limitation and values below −2 were called homogeneous selection.

To better understand the correlation between gut microbiota composition of the two species at different growth stages, a co-occurrence network was conducted based on Spearman correlation coefficients. In co-occurrence networks, nodes represent different genera levels. Gephi software (version 0.9.1) [46] undirected network with no direction at the edge and Fruchterman–Reingold layout were adopted. Different nodes in the network have different topological roles; thus, within-module connections (Zi) and inter-module connections (Pi) were used to describe the network topology, Zi describes the connections between its modules and other nodes, whereas Pi describes the connection between different modules. Based on previous research, co-occurrence networks were divided into four categories [47] according to different thresholds [48,49]: (1) module hubs, nodes with high connectivity inside the module (Zi > 2.5 and Pi < 0.62); (2) connectors, nodes with high connectivity between two modules (Zi < 2.5 and Pi > 0.62); (3) network hubs, nodes with high connectivity throughout the network (Zi > 2.5 and Pi > 0.62) and (4) peripherals, nodes with no high connectivity within and between modules (Zi < 2.5 and Pi < 0.62). The key nodes identified in the ecological co-occurrence network often represent key microbial species playing an important role in maintaining the stability of the microbial community structure [50].

### 2.6. Analysis of Predicted Functional Genes

Species composition information obtained from sequencing data was compared by using PICRUSt [51]. The databases of Kyoto Encyclopedia of Genes and Genomes (KEGG) [52] and Clusters of Orthologous Groups (COG) [53] were used to predict the function of OTUs. STAMP software [54] was used to infer the composition of functional genes in samples and analyze functional differences between different samples or groups. The differences with *p* values < 0.05 were considered statistically significant.

## 3. Results

### 3.1. Gut Microbiota Diversity

**Figure 1:**
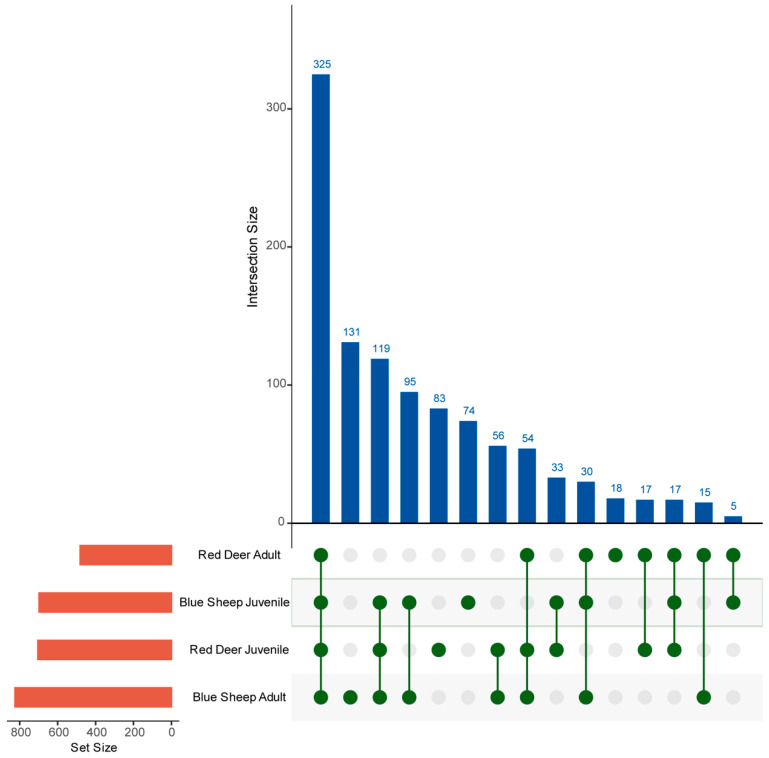
Core operational taxonomic units (OTUs) in the gut microbiome of captive blue sheep and red deer. Each dot refers to a sample group. Dots are visible below the graph, represent the number of core OTUs common to all microbial taxa and the proportion of each dot that does not overlap represents the number of specific OTUs.

**Figure 2:**
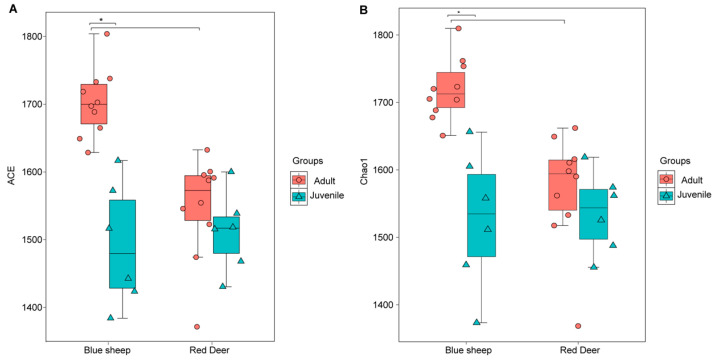
Dynamic changes in alpha-diversity metrics of the gut microbiota of captive blue sheep and red deer. (**A**) ACE index; (**B**) Chao1 index. All values are reported as mean ± SD, in which * indicates statistical significance (*p* < 0.05).

A total of 1,432,020 valid reads were obtained from fecal samples of blue sheep and red deer (Table S2). A total of 2303 OTUs were detected, among which 325 OTUs were shared by all sample groups, although different groups exhibited different microbiomes (Figure 1). ACE (*p* < 0.05) and Chao1 (*p* < 0.05) indices of the gut microbiota of juveniles were significantly lower than those of adults in blue sheep (Figure 2A,B). In red deer, at different growth stages, growth stages had no significant effect on gut microbiota richness and diversity (*p* > 0.05). In addition, ACE and Chao1 indices between species were significantly higher in adult sample groups compared to juvenile sample groups (Table 1).

**Figure 3:**
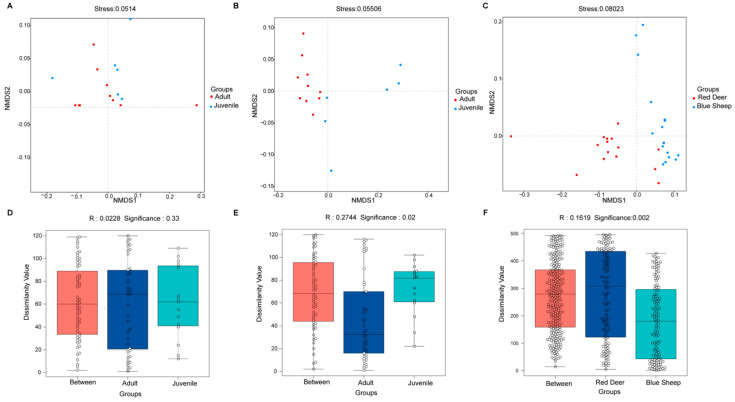
Combined analyses of non-metric multidimensional scaling (NMDS) (**A**–**C**) and Anosim (**D**–**F**) analyses of the gut microbiota of captive blue sheep and red deer based on unweighted Unifrac distance at the genus level. Anosim indicates significant differences between samples at different growth stages or not.

NMDS showed similarities in the structure of the gut microbiota based on weighted Unifrac at phylum and genus level. These results showed that the gut microbiota of adult and juvenile red deer did not differ significantly (Figure 3A,D). In contrast, the gut microbiota of blue sheep differed significantly among different growth stages (*p* < 0.05) (Figure 3B,E). Moreover, the gut microbiota of red deer and blue sheep was significantly different (*p* < 0.05) (Figure 3C,F).

### 3.2. Gut Microbiota Composition

**Figure 4:**
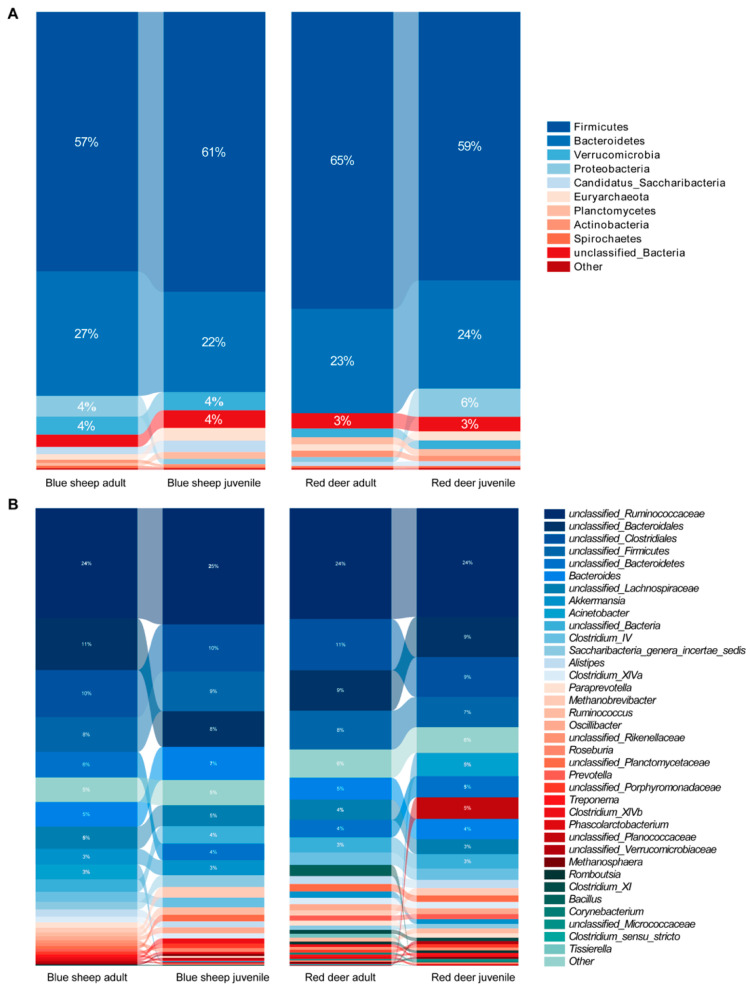
Composition of the gut microbiota of captive red deer and blue sheep at the (**A**) phylum and (**B**) genus level at different growth stages. Each percentage band indicates the ranking of bacterial species according to their relative abundance in each group.

According to the results of the phylogenetic classification of the gut microbiota of captive blue sheep and red deer, total sequences were divided into 25 phyla and 271 genera. The abundance of Firmicutes, Bacteroidetes, Verrucomicrobia, unclassified_Bacteria and Candidatus_Saccharibacteria accounted for over 95% of the gut microbiota in the two species. The composition of different groups of samples can be visualized at the gate level in Figure 4A. Firmicutes and Bacteroidetes were the two dominant phyla in the gut microbiota of red deer and blue sheep at different growth stages. In blue sheep, the proportion of Firmicutes in adults was lower than in juveniles, and Bacteroidetes in adults were higher than juveniles. However, it was the exact opposite in red deer. Considering inter-species differences, Firmicutes were higher in red deer than in blue sheep, and Verrucomicrobia were lower in red deer than blue sheep. The abundance of Candidatus_Saccharibacteria (*p* < 0.05) in the gut microbiota of blue sheep was significantly higher than that in the red deer. At genus level, the abundance of *unclassified_Clostridiales* and *unclassified_Firmicutes* in the gut microbiota of red deer is higher in adults than juveniles. In the gut microbiota of blue sheep, the abundance of *unclassified_Bacteroidales* significant increased (*p* < 0.05), representing 11.31% in adults and 7.97% in juveniles. Considering inter-species differences, the abundance of *Bacteroides* (6.14%) in blue sheep was significantly higher than that in red deer (4.7%) (Figure 4B). A heatmap of the top 37 genera based on Bray–Curtis dissimilarity was built, which shows the differences in relative abundance and clustering of dominant microbial genera in each group (Figure S2).

**Figure 5:**
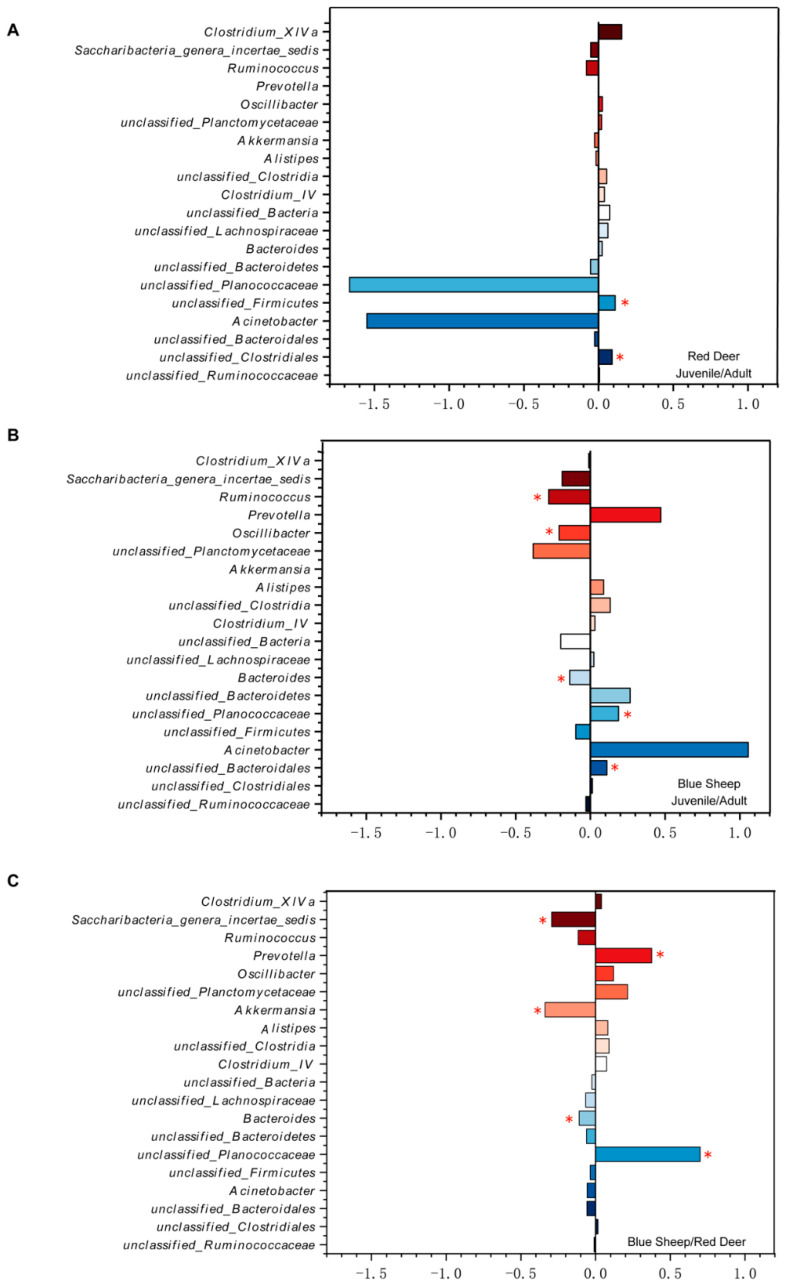
Differences in the trend in the top 20 genera in the gut microbiota of captive blue sheep and red deer in different growth stages. The y-axis represents the genus, and the x-axis represents the abundance of the genus at different growth stages or inter-species. (**A**) Red deer; (**B**) blue sheep; (**C**) inter-species (red deer and blue sheep) comparison. (* *p* < 0.05).

Using the Wilcox test at the genus level, we found inter-species differences in the composition and the relative abundance of bacteria in the gut microbiota. In addition, the relative abundance of *unclassified_Firmicutes* and *unclassified_Clostridia* were significantly different in red deer (*p* < 0.05) (Figure 5A). In blue sheep at different growth stages, the increase in the abundance of *Ruminococcus*, *Oscillibacter* were significantly different (*p* < 0.05) (Figure 5B). When considering inter-species differences, we found five genera were significantly different (Figure 5C). Notably, fewer differences were found in the composition of the gut microbiota of red deer in different growth stages.

**Figure 6:**
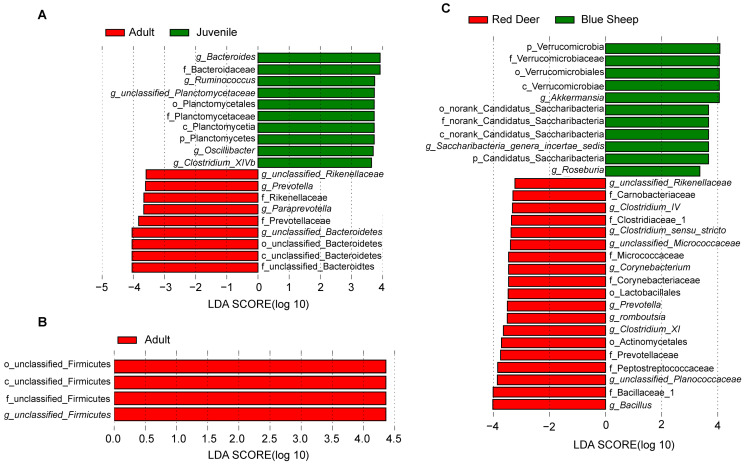
Distribution of linear discriminant analysis (LDA) scores. The ordinate indicates the taxon with significant differences between groups; the abscissa corresponds to the logarithmic LDA scores based on taxonomic analysis. (**A**) Differential microbial species in the gut microbiota of blue sheep; (**B**) differential microbial species in the gut microbiota of red deer; (**C**) differential microbial species in the gut microbiota between species.

Additionally, LEfSe analysis can enable determining biomarkers that are likely to be associated with differences between groups. Herein, differences in the gut microbiota of red deer in different growth stages were distributed within the phylum Firmicutes, which was also confirmed by the results of the Wilcox test. In contrast, a total of 19 potential biomarkers were found in blue sheep at different growth stages, which were mainly distributed within the phyla Firmicutes and Bacteroides (9 in the adults, 10 in the juveniles). Biomarkers belonged to the phylum Firmicutes in adult red deer, and 30 potential biomarkers at the inter-species level (11 in the blue sheep group, 19 in the red deer group). An increased number of distinct microbial taxa were identified in the inter-species analysis. *Bacteroides*, *Ruminococcus* and *Oscillibacter* were the most abundant genera in juvenile blue sheep (Figure 6A). *Unclassified_Firmicutes* was the most abundant genus in blue sheep (Figure 6B). Between species, *Akkermansia* and *Roseburia* were the most abundant genera in blue sheep; *Bacillus*, *unclassified_Planococcaceae* and *Clostridium_XI* were the most abundant genera in red deer (Figure 6C). The previously discussed blue sheep show significant differences at different growth stages and between species in NMDS. *Oscillibacter* may be a potential marker to distinguish the gut microbiota composition of blue sheep at different growth stages. *Akkermansia* may be potential markers between species in this study. Furthermore, cladograms were constructed to demonstrate phylogenetic differences in gut microbial composition across growth stages and species (Figure S3).

### 3.3. Gut Microbiota Community Assembly and Co-Occurrence Network Analysis

**Figure 7:**
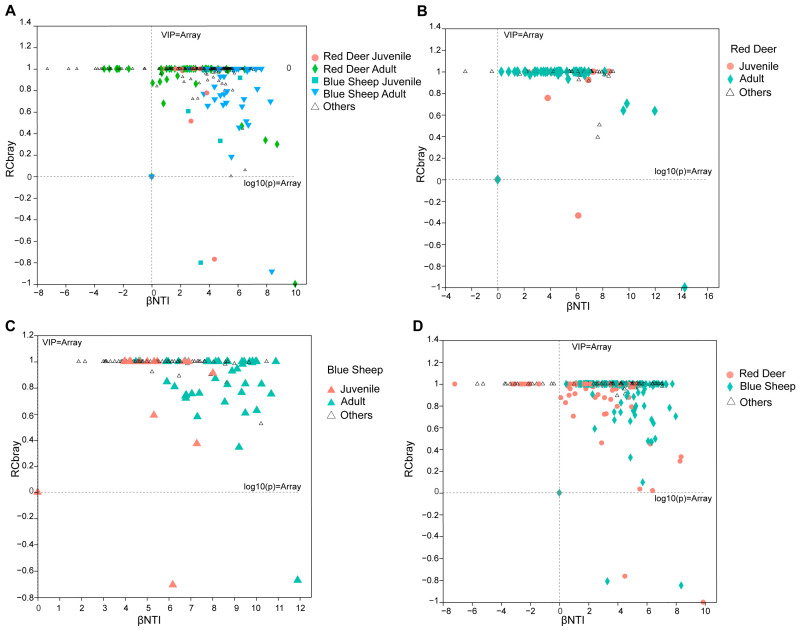
Community structure of the gut microbiota of captive blue sheep and red deer based on βNTI and RCbray calculations to indicate randomness or determinism models in the microbial community assembly. (**A**) βNTI/RCbray community structure analysis of all groups; (**B**) community structure analysis of the gut microbiota of red deer; (**C**) community structure analysis of the gut microbiota of blue sheep; (**D**) community structure analysis of the gut microbiota between species.

Subsequently, βNTI values were calculated for captive red deer and blue sheep in different growth stages, as well as for inter-species samples, to quantify the relative contributions of randomness versus determinism to the assembly of the microbial community structure of the gut microbiota of red deer and blue sheep. To determine the influencing factors between different sample groups, the relative importance of bacterial community assembly method (deterministic and stochastic) was calculated among different growth stages and species by determining βNTI/RCbray values (Figure 7A). Certain βNTI/RCbray values of community structure of the gut microbiota of red deer at different growth stages were between −2 and 2, but most values were greater than 2, which was a type of heterogeneous selection (Figure 7B). In contrast, the distribution of βNTI values of most samples for blue sheep at different growth stages was greater than 2 (Figure 7C). Thus, it could be demonstrated that differences in the gut microbiota of blue sheep and red deer were caused by factors related to the developmental stage. In contrast, when considering inter-species comparisons, the distribution range of βNTI values of most samples was greater than 2, indicating that differences between the gut microbiota of blue sheep and red deer were caused by factors related to species type (Figure 7D). Thus, differences in the microbial community of the gut microbiota of captive blue sheep and red deer among different growth stages and inter-species comparisons were due to deterministic factors.

**Figure 8:**
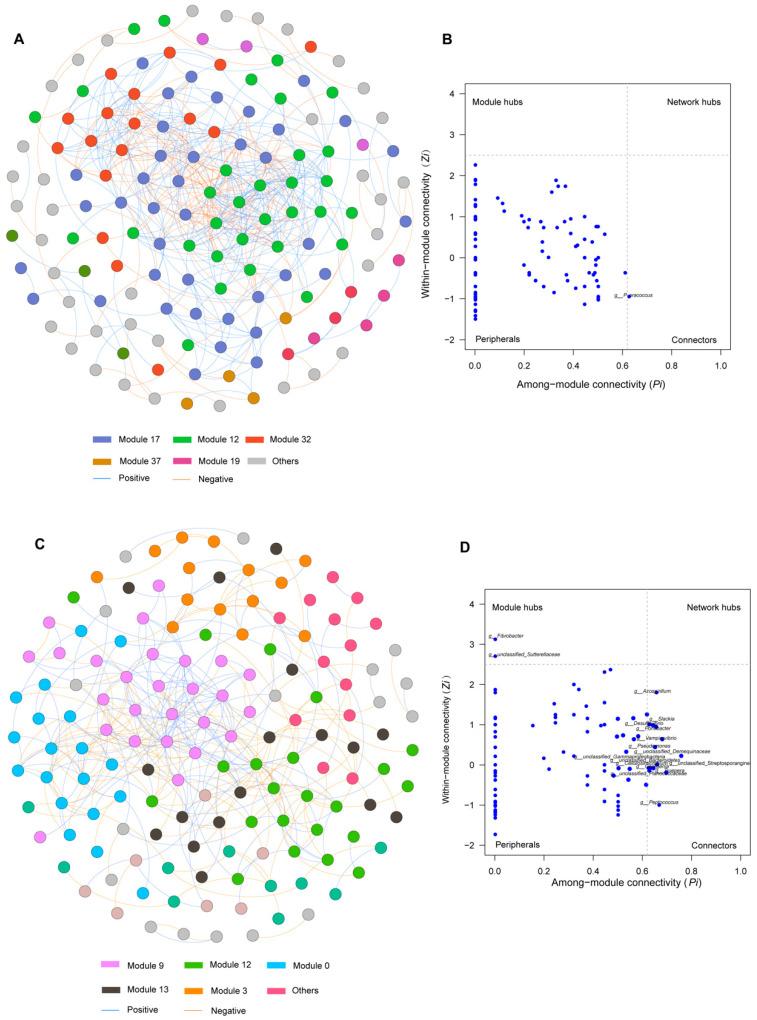
Co-occurrence network analysis of gut microbiota communities in captive red deer (**A**) and blue sheep (**C**). Zi-Pi map based on node features of the gut microbiota community of red deer (**B**) and blue sheep (**D**). The blue edge represents a positive correlation; the yellow edge represents a negative correlation. Each circle or node represents a genus, node colors indicate different microbial communities.

To identify interactions between the gut microbiota of blue sheep and red deer, we constructed a co-occurrence network; all generated networks are modular. The number of significantly correlated nodes in red deer is high at the genus level (501), while the number of blue sheep sample nodes is small (362) (Figure 8A,C). The larger number of nodes in red deer also indicates a tighter co-occurrence network; mutual cooperation between microorganisms was relatively loose in blue sheep. Red deer and blue sheep obtained 37 and 13 functional modules, respectively. In addition, co-occurrence networks also revealed correlations between microbial taxa and functions, with more positive than negative correlations found in red deer than in blue sheep. In general, the network structures between blue sheep and red deer and the nodes between them are quite different.

Furthermore, two important node features are derived from network analysis modules: within-module connectivity (Zi) and inter-module connectivity (among-module connectivity, Pi), which were constructed into ZP diagrams based on these two properties (Figure 8B,D). In particular, module hubs and connectors were highly linked to multiple microbial species within the respective modules and were, therefore, considered to be the most representative key microorganisms of the gut microbiota of blue sheep and red deer. In red deer, only one key bacterium, *Paracoccus,* was identified and found in connector hubs in module 32, whereas the rest of the nodes belonged to peripherals, indicating the absence of a high degree of connection within or between modules. In blue sheep, two key microbial nodes were found, i.e., *Fibrobacter* in module 3 and *unclassified_Sutterellaceae* in module 12, and key microorganisms established highly connected nodes inside the module; in addition, 15 microorganisms were found in connectors, thus indicating a high degree of connection among multiple modules (Table 2).

### 3.4. Functional Prediction of the Gut Microbiome of Captive Blue Sheep and Red Deer

**Figure 9:**
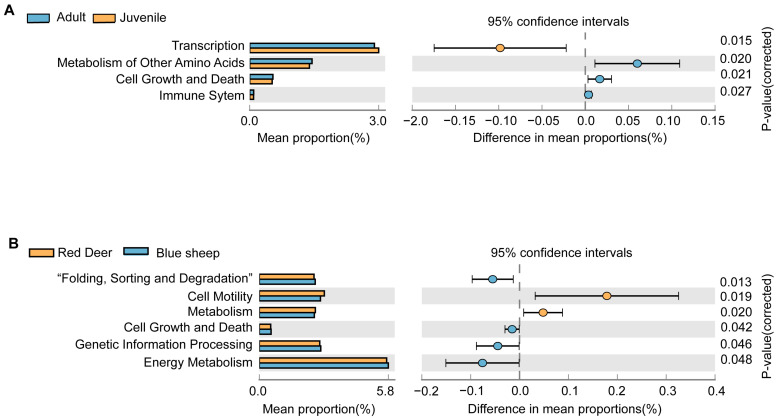
KEGG metabolic pathway analysis between second-level categories. (**A**) Comparison of the gut microbiota of juvenile and adult blue sheep; (**B**) Inter-species comparison of the functions of the gut microbiota. Different colors indicate different groups. On the left side of the figure is shown the abundance ratio of different functions in the two sample groups; in the middle is shown the different rate of function abundance with a 95% confidence interval; on the right side, *p* values are reported.

Based on the KEGG metabolic pathway analysis conducted with PICRUSt software, the function of the gut microbiome of captive blue sheep and red deer in different growth stages and inter-species differences was compared. Considering the top 10 most enriched KEGG metabolic pathways, the five most important metabolic pathways in samples of different growth stages and species groups were membrane transport, carbohydrate metabolism, amino acid metabolism, replication and repair and translation. No significant differences were found in the function of the gut microbiota in red deer at different growth stages. In the gut microbiota of blue sheep at different growth stages, the four most significantly enriched KEGG pathways were transcription, metabolism of other amino acids, cell growth and death and immune system. Among these, transcription was more enriched in adult blue sheep, and the remaining three pathways were more abundant in juvenile blue sheep (Figure 9A). Considering inter-species comparisons, cell motility and metabolism were more enriched in red deer compared to blue sheep, while the remaining four pathways were more abundant in blue sheep, while folding, sorting and degradation, cell growth and death, genetic information processing and energy metabolism were the four pathways more abundant in blue sheep (Figure 9B).

**Figure 10:**
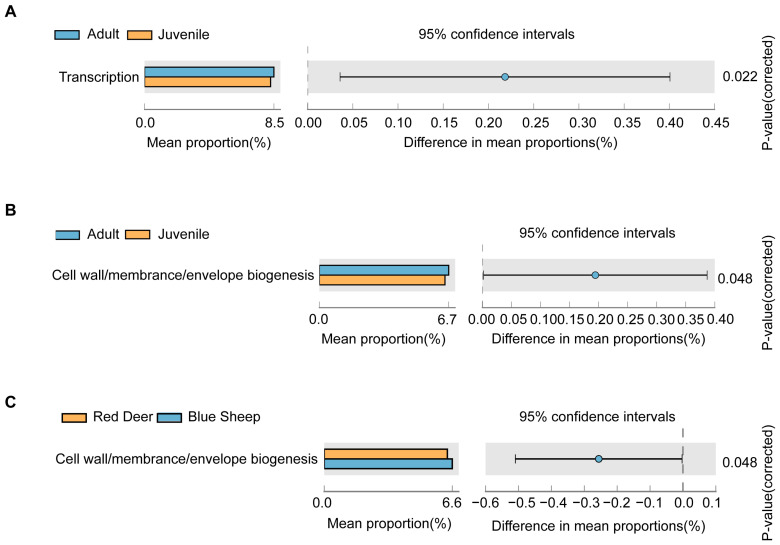
Clusters of Orthologous Groups (COG) functional analysis of the gut microbiome of captive blue sheep and red deer. (**A**) Comparison between juvenile and adult red deer; (**B**) comparison between juvenile and adult blue sheep and (**C**) comparison between blue sheep and red deer.

The Clusters of Orthologous Groups (COG) database enables protein functional classification for prokaryotes. Based on the COG database, the top 22 most enriched metabolic pathways were investigated. In particular, the category transcription was found to be differently enriched in red deer at different growth stages (Figure 10A). However, significant differences were found in the category of cell wall/membrane/envelope biogenesis in blue sheep at different growth stages (Figure 10B). Moreover, cell wall/membrane/envelope biogenesis was found to be more abundant in blue sheep upon inter-species comparison (Figure 10C).

## 4. Discussion

The gut microbiota are a dynamic ecosystem, and it can be assumed that different growth stages might have certain effects on their structure and composition. Therefore, understanding the factors that contribute to gut microbiota diversity and composition at different life stages is critical. The present study explored gut microbial composition and functions in captive blue sheep and red deer. Using *16S rRNA* gene sequencing technology, it was found that different growth stages and inter-species comparisons have differences in gut microbiota composition.

### 4.1. Associations of Gut Microbiota with Growth Stages

Microbial diversity across growth stages correlates with growth needs. Previous studies have shown that the diversity of the mammalian gut microbiota increases with age until its composition becomes stable [10,55]. This is consistent with our finding that adult blue sheep have higher gut microbiota richness than juveniles. However, no significant difference was found in alpha-diversity metrics of the gut microbiota in red deer at different growth stages, which may be due to the age proximity between the juvenile and adult red deer evaluated herein. In particular, the lower diversity in the gut microbial communities of juvenile populations may be due to compromised resistance to colonization, whereas with age, stable microbial communities gradually form. Based on NMDS analysis, differences in the composition of the gut microbiota of blue sheep and red deer at different growth stages were observed. Moreover, Anosim analysis showed that, at the genus level, differences in the gut microbiota of red deer in different growth stages were not significant, which may be caused by the age proximity of the evaluated individuals, which, however, requires further research.

In addition, the results described herein showed that Bacteroides were more abundant in the gut microbiota of captive blue sheep, especially in the adult stage of development. This may be related to environmental conditions and stable feed intake adopted in captivity. In contrast, Firmicutes and Bacteroides were more abundant in the gut microbiota of red deer, with a small amount of spirochetes, which were similar to the results of previous studies [17]. Previous studies have shown that the microbial community maintains a certain stability and consistency within the host considering the same time and space [56,57,58], which is in line with the findings of our study that, with the increase in age, the abundance of microorganisms in the gut microbiota in red deer and blue sheep showed a certain difference, but dominant phyla were nevertheless Firmicutes and Bacteroidetes. Collectively, this indicated that different growth stages affected the composition of the gut microbiota of red deer and blue sheep, but the dominant phyla in the respective microbiota did not change.

The species richness and structure of the gut microbiota usually reflect the health status of the body. Moreover, it has been demonstrated that diseases may occur by abnormalities in the gut microbiota, and certain microbial species might contribute to the occurrence of certain intestinal disorders [59]. The gut microbiota of red deer and blue sheep were mainly enriched by *unclassified_Ruminococcaceae*, *unclassified_Clostridiales* and *unclassified_Bacteroidales*. These bacteria can degrade fibers and produce organic acids and SCFAs [60]. Among these, members of the family Bacteroides can instantly produce butyric acid, degrade plant polysaccharides and improve nutrient utilization [61,62,63]. In the present study, the abundance of certain microbial species changed significantly with age in the gut microbiota of red deer and blue sheep. For instance, the abundance of Firmicutes (*unclassified_Ruminococcaceae*, *unclassified_Clostridiales, unclassified_Planococcaceae*, *Treponema, Ruminococcus*, *Clostridium_XlVb*, *Roseburia*), Bacteroides (*unclassified_Bacteroidales*, *unclassified_Bacteroidetes*, *Bacteroides*, *unclassified_Bacteria*, *Paraprevotella*) and *Saccharibacteria_genera_incertae_sedis*. Most of these bacteria are related to the adaptive immune system, in which health and the intestinal environment likely play a key role. However, in the present study, *Alistipes* [64], *unclassified_Clostridia* and *Oscillibacter* were among the dominant bacterial genera in the gut microbiota of red deer and blue sheep, and both types of ungulates may suffer from colon or metabolic disorders [65], premature aging [66] and increased risk of cardiovascular disease [67,68]. However, an increase in the abundance of *Bacillus*, *Clostridium_sensu_stricto* and *unclassified_Clostridia* may negatively affect red deer health [69,70]. Most of the genera were unclassified microorganisms, which may be due to the wide variety of microorganisms that have not yet included in the database and require further future studies.

Most gut microbial species are beneficial to the host, promote the development of gut-associated lymphoid tissue, and are an important antigen. The results of LEfSe analysis showed that most of the potential biomarkers in the gut microbiota of red deer at different growth stages were distributed within the phylum Firmicutes, while biomarkers of the gut microbiota of blue sheep were mainly distributed within the phyla Firmicutes, Planctomyces and Bacteroides. It is known that when Firmicutes and Bacteroides are found in high abundance, the host’s food digestion process can be facilitated and contribute to energy intake [71,72,73]. In adult blue sheep, *unclassified_Bacteroidales* improved nutrient availability and prevented disease, whereas *Prevotella* can decompose plant polysaccharides and host-derived mucins [74]. Moreover, *unclassified_Rikenellaceae* has the function of immune modification and inhibition of inflammatory cytokines. *Oscillibacter* and *Clostridium_XIVb* had a certain impact on host health in red deer and blue sheep. The abundance of *Oscillibacter* in juveniles was greater than that in adults of blue sheep, with significant differences, which can produce unpleasant-smelling valeric acid by anaerobic bacteria to stimulate the intestinal mucosa [75], thus affecting the host’s intestinal health [76]. This may be related to the different strategies adopted for the two species to different environmental conditions at different growth stages; however, further studies are needed to confirm this speculation [77].

In co-occurrence network analysis, the key bacterial species in the gut microbiota of red deer was *Paracoccus*. Among these, *Fibrobacter* in module 3 of blue sheep was able to decompose cellulose [78], whereas *unclassified_Gammaproteobacteria* and *Cellulosimicrobium* can balance intestinal health, thus having an antibacterial effect [79]. Moreover, *Peptococcus* and *unclassified_Bacteroidetes* have the function of fermentation metabolism and disease prevention [80], and most of these bacterial genera are closely related to animal health. The functions of some core bacterial genera have not been completely determined, which may be due to the current limited database; hence, this requires further verification in future studies.

By comparing KEGG enriched pathways in the gut microbiota of captive blue sheep and red deer, differences in the functions of the gut microbiota of red deer in different growth stages were not significant, which may be due to a similar composition of the gut microbiota. Moreover, differences in gut function and immune function between different growth stages were revealed in COG analysis.

### 4.2. Associations of Gut Microbiota with Inter-Species

We found that the dominant phyla (over 80% relative abundance) in the inter-species gut microbiota were Firmicutes and Bacteroidetes, which agreed with the findings of previous reports on the composition of the gut microbiota of herbivorous animals [81,82]. Herein, environment and dietary factors were carefully controlled; hence, significant differences found in the present study in the composition of the gut microbiota are more likely to be related to developmental stage and animal species. In contrast, significant differences were found in ACE and Chao1 indices between adult blue sheep, which may be due to differences in the regulatory ability and adaptability between species [83].

In inter-species LEfSe analysis, *Akkermansia* found in the gut microbiota of blue sheep can promote intestinal barrier integrity and inhibit inflammation [84]. *Roseburia* is mostly distributed in the cecum, and the remaining biomarker microorganisms in the gut microbiota mainly produced butyrate [85]. In the gut microbiota of red deer, *unclassified_Rikenellaceae, Prevotella, Romboutsia, Clostridium_XI* and *Bacillus* were found to be associated with modified immune function in red deer. *Clostridium_IV* is extremely resistant to adverse environmental conditions [86], whereas *Prevotella* can lower cholesterol levels and consume dietary fiber [87]. Thus, understanding the relationship between opportunistic pathogens existing among species can provide relevant basic information for the prevention and control of species epidemics, and several inflammatory conditions are caused by imbalances in the gut microbiota.

Moreover, the number of genes in the gut microbiome exceeds greatly that of the host genome; thus, the gut microbiome is considered important to promote health and participate in the construction of the gut micro-ecosystem [88]. A previous study showed that the composition of the gut microbiota is related to the immune system status and health of the host [89]; hence, the results observed in the present study may indicate differences in immune function between species.

More detailed information on the gut microbiota of captive red deer and blue sheep could enlarge the current understanding of the health status of the species. In future studies, mutual utilization and competition between bacterial species in the gut microbiota of captive red deer and blue sheep should be further considered, which will help to better understand changes in the gut microbiota diversity of ungulates in different growth stages, which has important biological value and significance for guiding ungulate diet structure, hence preventing intestinal diseases and improving overall health level. The results discussed herein provide a basis for captivity management of wild animals, thereby supporting the design of improved reintroduction plans of captive animals.

## 5. Conclusions

In the present study, differences in the gut microbiota of ungulates at different growth stages and between species were studied. The diversity and composition of the gut microbiota of captive blue sheep and red deer at different growth stages changed, but the composition of dominant microbial phyla in the gut microbiota remained stable. Moreover, several immune-related microbial communities and some pathogenic bacterial species were also found. Thus, predicting pathogenic communities in the gut microbiota can effectively help hosts adapt more rapidly to environmental conditions found in captivity. Furthermore, functional predictions of the gut microbiota revealed differences in the functional characteristics in blue sheep and red deer at different growth stages and between the two species. Future research will focus on determining the physiological role of these distinct microorganisms in regulating different growth stages or across species. Finally, the relationship between different growth stages of captive ungulates and the composition of gut microbiota was explored in the current work, thus providing important information for the formulation of health protection strategies, intestinal disease treatment programs and dietary structure adjustments for captive ungulates at different growth stages.

## Figures and Tables

**Table 1:** Shannon ACE Chao1 Simpson phylogenetic diversity of populations at different growth stages.

Group	Growth Stage	Shannon	ACE	Chao1	Simpson
Red Deer	Juvenile	5.48 ± 0.42	1547.36 ± 77.07	1570.67 ± 0.001	0.02 ± 0.02
	Adult	5.28 ± 0.73	1511.23 ± 58.61	1537.32 ± 59.79	0.03 ± 0.05
*p* value	2 groups	*p* > 0.05	*p* > 0.05	*p* > 0.05	*p* > 0.05
Blue Sheep	Juvenile	5.56 ± 0.21	1702.20 ± 50.28	1719.28 ± 45.74	0.01 ± 0.003
	Adult	5.39 ± 0.21	1491.96 ± 91.19	1527.03 ± 102.27	0.01 ± 0.003
*p* value	2 groups	*p* > 0.05	*p* < 0.05	*p* < 0.05	*p* > 0.05

**Table 2:** Topological role shift between different growth stages in blue sheep and red deer.

Groups	Correlation	Genera	No. Module	Network Roles
Red deer	0.48	*Paracoccus*	3	Connectors
Blue sheep	0.52	*Azospirillum*	6	Connectors
		*Cellulosimicrobium*	8	Connectors
		*Desulfovibrio*	9	Connectors
		*Fibrobacter*	1	Module hubs
		*Georgenia*	8	Connectors
		*Peptococcus*	5	Connectors
		*Pontibacter*	7	Connectors
		*Pseudomonas*	4	Connectors
		*Slackia*	4	Connectors
		*Truepera*	3	Connectors
		*Vampirovibrio*	3	Connectors
		*unclassified_Bacteroidetes*	5	Connectors
		*unclassified_Demequinaceae*	3	Connectors
		*unclassified_Gammaproteobacteria*	8	Connectors
		*unclassified_Planococcaceae*	3	Connectors
		*unclassified_Sutterellaceae*	3	Module hubs

## Data Availability

All *16S rRNA* gene sequences obtained in the present study are available in the NCBI database under the bioproject number PRJNA825498.

## Supplementary Materials

The following supporting information can be downloaded at: https://www.mdpi.com/article/10.3390/ani13040553/s1, Table S1: Sampling animal information; Figure S1: The living environment of blue sheep (**A**) and red deer (**B**) in different growth stages; Table S2: Statistical table of sequencing quantity of sample; Figure S2: Ranking of the abundance of the top 37 bacterial genera in the gut microbiota of captive blue sheep and red deer based on a Bray–Curtis dissimilarity heatmap. The relative percentage of each bacterial genus (variable clustered on the y-axis) in each sample is primarily depicted; Figure S3: Taxonomic clade diagram indicating the phylogenetic distribution of microbial lineages associated with each group. In each group, the difference is indicated by a unique color. Circles represent the phylogenetic level from phylum to genus (OTUs) from the inside out, and the diameter of each circle is proportional to the abundance of taxon. (**A**) Juvenile and adult blue sheep; (**B**) Juvenile and adult red deer; (**C**) Blue sheep and red deer. Video S1: Blue sheep in the semi-enclosed environment of Zhongshan Park, China.
