# Supplementary material for: Growth Stages and Inter-Species Gut Microbiota Composition and Function in Captive Red Deer (Cervus elaphus alxaicus) and Blue Sheep (Pseudois nayaur)
Source: Animals (Basel). 2023 Feb 4;13(4):553. doi: 10.3390/ani13040553 (PMC9951700; doi:10.3390/ani13040553)
Supplement: Supplementary file 1 [file animals-13-00553-s001.zip › animals-2126266-Supplementary file.pdf]

Developmental Stages Influence Gut Microbiota Composition and Function of Captive Red Deer (*Cervus elaphus alxaicus*) and Blue Sheep (*Pseudois nayaur*)

Yao Zhao [1], Jia Sun [2], Mengqi Ding [3], Romaan Hayat Khattak [1,4], Liwei Teng [1,5,*] and Zhensheng Liu[1,5,*]

[1]College of Wildlife and Protected Areas, Northeast Forestry University, Harbin 150040, China

[2] Liaoning Wildlife Protection and Epidemic Disease Monitoring Center, Dalian 116013, China

[3] State Key Laboratory of Urban Water Resource and Environment, School of Environment, Harbin Institute of Technology, Harbin 150090, China

[4] Institute of Zoology, Guangdong Academy of Sciences, Guangzhou 510260, China

[5] Key Laboratory of Conservation Biology, National Forestry and Grassland Administration, Harbin 150090, China

* Correspondence: tenglw1975@163.com (L.W.T.); zhenshengliu@163.com (Z.S.L.)

Table S1. Sampling animal information

Sample ID	Sex	Growth stage	Weight（kg）	Age（year）
ML1	Male	Adult	150	10
ML2	Male	Adult	140	8
ML3	Male	Adult	130	5
ML4	Male	Adult	120	4
ML5	Male	Adult	150	7
ML6	Female	Adult	100	9
ML7	Female	Adult	105	8
ML8	Female	Adult	125	6
ML9	Female	Adult	130	8
ML10	Female	Adult	120	4
ML1-y	Male	Juvenile	70	2
ML2-y	Male	Juvenile	65	2
ML3-y	Male	Juvenile	65	2
ML4-y	Female	Juvenile	65	2
ML5-y	Female	Juvenile	65	2
ML6-y	Female	Juvenile	65	2
YY1	Male	Adult	30	9
YY2	Male	Adult	30	5
YY3	Male	Adult	22.5	4
YY4	Male	Adult	22.5	3
YY5	Male	Adult	20	12
YY6	Female	Adult	21	11
YY7	Female	Adult	20	10
YY8	Female	Adult	20.5	5
YY9	Female	Adult	19	3
YY10	Female	Adult	15	1.3
YY1-y	Male	Juvenile	9	1.3
YY2-y	Male	Juvenile	9	1.3
YY3-y	Male	Juvenile	9	0.3
YY4-y	Female	Juvenile	8.5	0.3
YY5-y	Female	Juvenile	8.5	0.3
YY6-y	Female	Juvenile	8	0.3

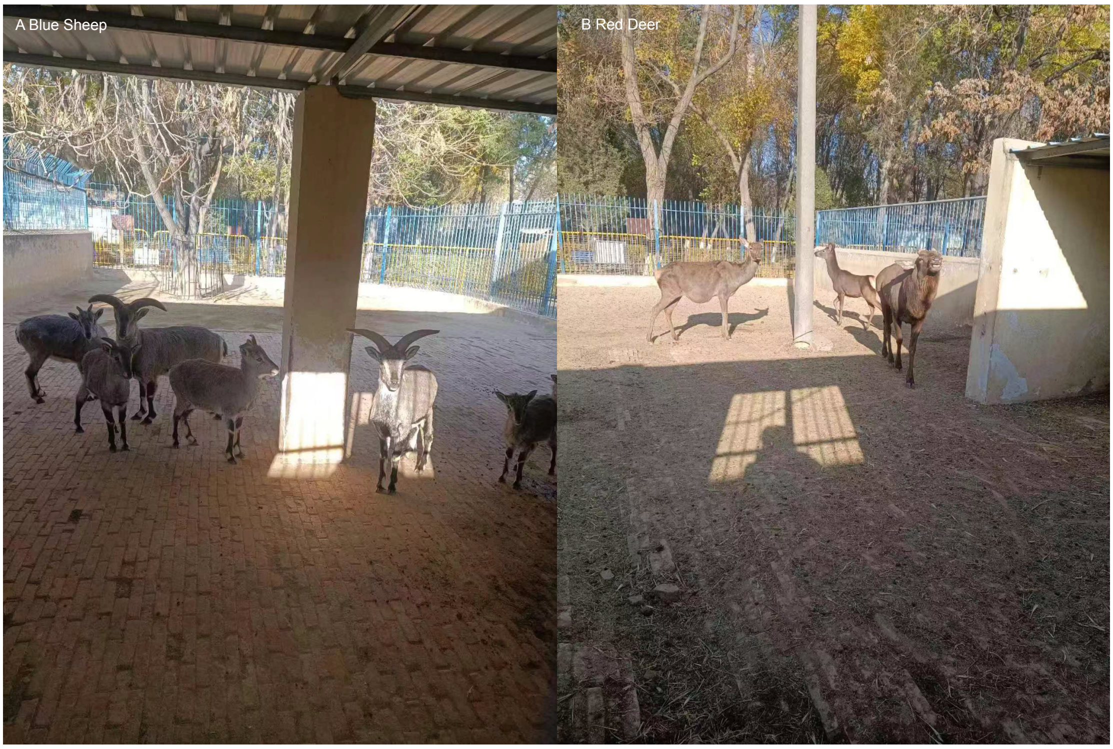

Figure S1. The living environment of blue sheep (**A**)and red deer(**B**) in different growth stages.

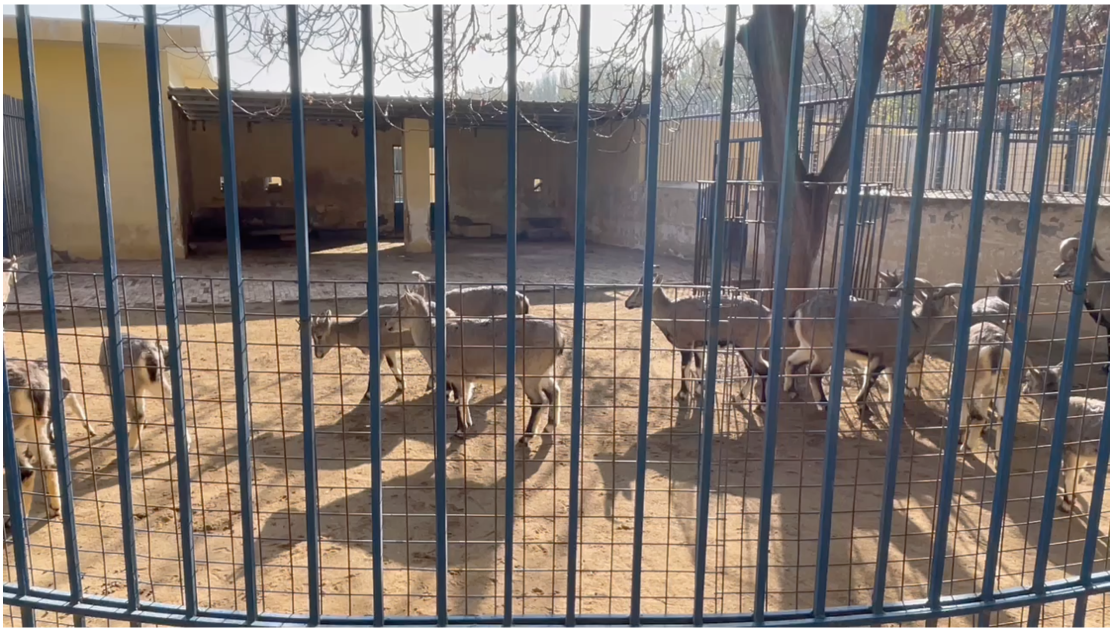

Video S1. Blue sheep in the semi-enclosed environment of Zhongshan Park, China.

Table S2. Statistical table of sequencing quantity of sample

Red Deer	Sequencing quantity	Effective sequencing quantity	Blue Sheep	Sequencing quantity	Effective sequencing quantity
ML1	75939	75463	YY1	70509	70002
ML2	58924	58784	YY2	77965	77663
ML3	79273	78944	YY3	89270	88662
ML4	77734	77376	YY4	78511	78012
ML5	73570	72778	YY5	87868	86885
ML6	66538	65677	YY6	74404	74017
ML7	56697	56304	YY7	71962	71322
ML8	69223	68756	YY8	64170	63812
ML9	81108	80599	YY9	69904	69278
ML10	81752	81329	YY10	83361	82812
ML1-y	79750	79070	YY1-y	78713	77946
ML2-y	67034	66643	YY2-y	64476	63936
ML3-y	61477	61173	YY3-y	75641	75188
ML4-y	75476	75015	YY4-y	92502	91461
ML5-y	80991	80590	YY5-y	75926	75345
ML6-y	74566	74260	YY6-y	89109	88771

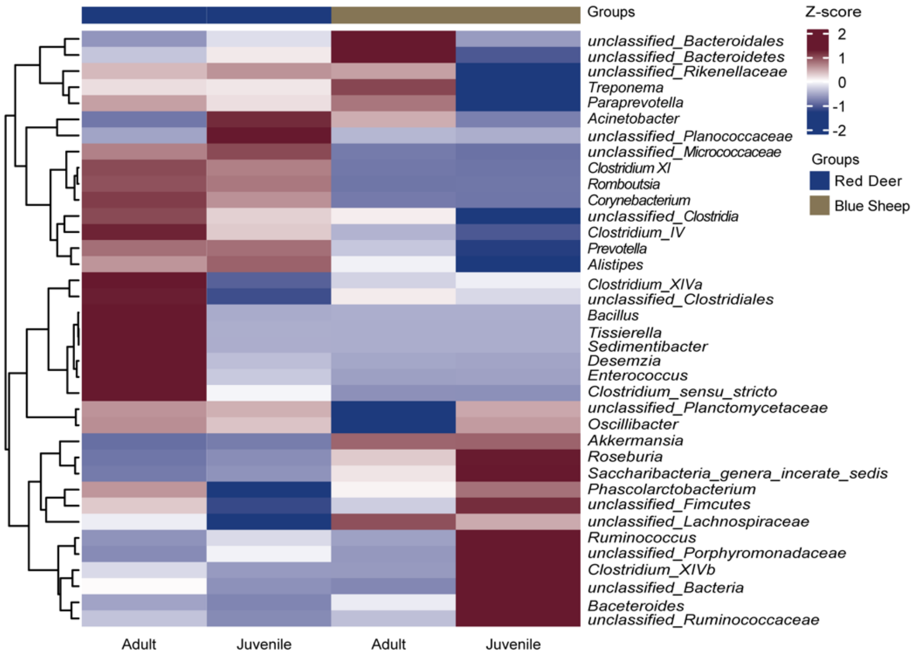

Figure S2. Ranking of the abundance of the top 37 bacterial genera in the gut microbiota of captive blue sheep and red deer based on a Bray-Curtis dissimilarity

heatmap. The relative percentage of each bacterial genus (variable clustered on the y-axis) in each sample is primarily depicted.

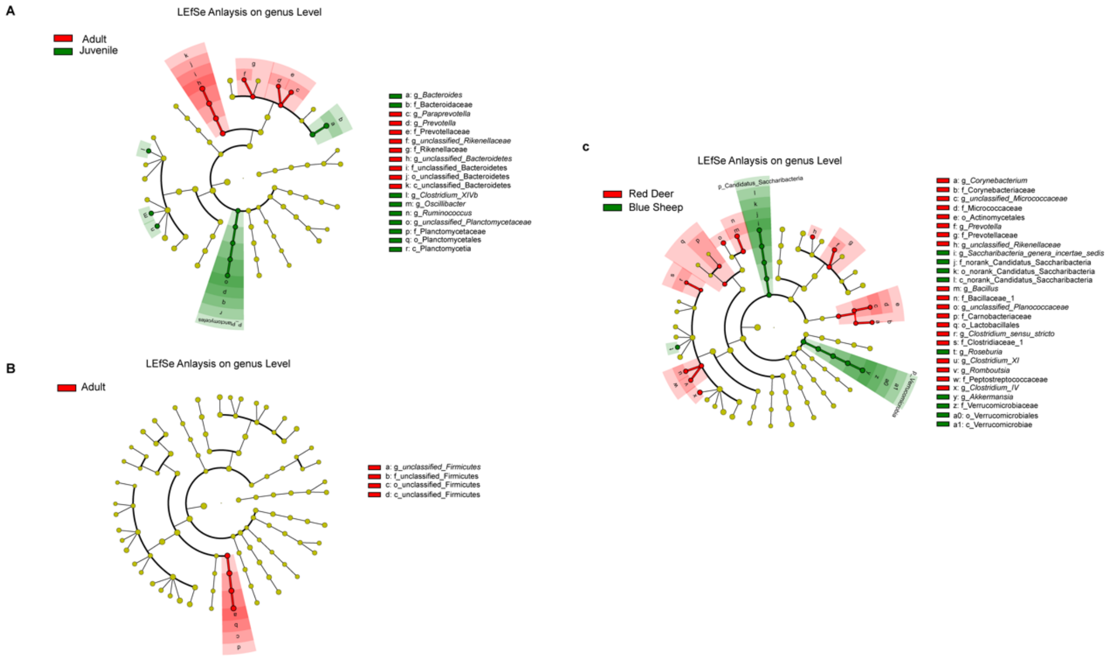

Figure S3. Taxonomic clade diagram indicating the phylogenetic distribution of microbial lineages associated with each group. In each group, the difference is indicated by a unique color. Circles represent the phylogenetic level from phylum to genus (OTUs) from the inside out, and the diameter of each circle is proportional to the abundance of taxon. (**A**) Juvenile and adult blue sheep; (**B**) Juvenile and adult red deer; (**C**) Blue sheep and red deer.
